# Metabolic permissiveness: how tissue context shapes cancer

**DOI:** 10.1101/gad.353516.125

**Published:** 2026-03-01

**Authors:** Chrysanthi Moschandrea, Christian Frezza

**Affiliations:** 1 Faculty of Medicine and University Hospital Cologne, Institute for Metabolomics in Ageing, Cluster of Excellence Cellular Stress Responses in Aging-Associated Diseases (CECAD), University of Cologne, 50931 Cologne, Germany;; 2 Faculty of Mathematics and Natural Sciences, Institute of Genetics, Cluster of Excellence Cellular Stress Responses in Aging-Associated Diseases (CECAD), University of Cologne, 50931 Cologne, Germany;; 3 Center for Molecular Medicine (CMMC), University of Cologne, 50931 Cologne, Germany

**Keywords:** cancer, metabolism, permissiveness

## Abstract

An emerging paradox in cancer metabolism is that identical oncogenic mutations produce profoundly different metabolic phenotypes depending on tissue context, with many mutations exhibiting striking tissue-restricted distributions. Here we introduce metabolic permissiveness as the inherent capacity of a tissue to tolerate, adapt to, or exploit metabolic disruptions, providing a unifying framework for explaining this selectivity. We examine tissue-specific metabolic rewiring driven by canonical oncogenes (MYC and KRAS), tumor suppressors (p53, PTEN, and LKB1), and tricarboxylic acid (TCA) cycle enzymes (FH, SDH, and IDH), demonstrating that baseline metabolic architecture, nutrient microenvironment, redox buffering, and compensatory pathways determine whether mutations confer a selective advantage or metabolic crisis. We further discuss how the tumor microenvironment shapes metabolic adaptation and therapeutic vulnerability. This framework reveals shared principles of tissue-specific metabolic vulnerability in cancer and provides a mechanistic basis for precision metabolic therapies.

## Abstract (precis)

In this review, Moschandrea and Frezza discuss the tissue-specific metabolic outcomes of canonical oncogene activation, aberrant tumor suppressor function, or mutated TCA cycle genes. Together with tissue-specific tumor microenvironments, they posit that the inherent metabolic permissiveness of a tissue environment shapes the metabolic adaptation of cancer cells and could have implications for precision medicine.

Cancer is a complex group of diseases characterized by uncontrolled cell proliferation and, often, the capacity to invade surrounding tissues and metastasize to distant organs (Weinberg 1996). A series of molecular alterations drives this malignant transformation, as described in the “hallmarks of cancer” (Hanahan and Weinberg 2011; Pavlova and Thompson 2016; Hanahan 2022). Among these, the reprogramming of cellular metabolism has emerged as a central feature, enabling cancer cells to sustain rapid growth, resist metabolic and oxidative stress, and modulate the tumor microenvironment in ways that favor survival and disease progression—not through a universal increase in energy production but by a redistribution of metabolic fluxes toward biosynthetic, redox, and stress-adaptive pathways (Hanahan and Weinberg 2011; Bartman et al. 2023). Although metabolic reprogramming was only recently added to the formal list of cancer hallmarks, the concept that tumor cells exhibit altered nutrient handling has been recognized for more than a century. Early clinical and experimental studies reported elevated blood glucose levels in cancer patients, linked tumor growth to dietary carbohydrate intake, and observed altered oxygen consumption in malignant proliferation (Freund 1885; von Wassermann et al. 1911; Van Alstyne and Beebe 1913; Woglom 1915). The seminal discovery by Otto Warburg (1924, 1956) that cancer cells preferentially convert glycolysis to lactate even in the presence of sufficient oxygen, a phenomenon known as aerobic glycolysis or the Warburg effect, laid the foundation for thinking about cancer as a metabolic disease. Warburg (1924, 1956) proposed that defective mitochondrial respiration was the primary cause of malignancy. However, his hypothesis was largely overshadowed by the emergence of molecular oncology, which shifted the focus toward oncogenes, tumor suppressors, and intracellular signaling cascades as the main drivers of cancer development. Intriguingly, however, it was eventually shown that mutations in genes such as *MYC*, *KRAS*, and *PIK3CA*, as well as loss of tumor suppressors like *TP53* and *LKB1* (Edinger and Thompson 2002; Shackelford and Shaw 2009; Vousden and Ryan 2009; Dang 2012; Ying et al. 2012), not only have oncogenic functions but also orchestrate the previously observed metabolic changes. The subsequent discovery in the early 2000s of recurrent mutations in core metabolic enzymes such as isocitrate dehydrogenase (IDH), succinate dehydrogenase (SDH), and fumarate hydratase (FH) provided definitive evidence that deregulated metabolism is not merely an epiphenomenon of oncogenic signaling but can itself drive malignant transformation.

While initially considered a universal reprogramming with specific pathways being essential, recent findings enabled by advances in metabolomics approaches revealed that oncogenic metabolic rewiring is exquisitely dependent on the tissue of origin and the stage of tumor progression (Yuneva et al. 2012; Davidson et al. 2016; Hensley et al. 2016). The same oncogenic mutation can yield markedly different effects across tissues depending on their intrinsic metabolic state and may drive tumorigenesis in one organ while having little to no impact in another. These observations demonstrate that metabolic reprogramming is not determined solely by genetic alterations but by the interplay between oncogenic mutations and the tissue's intrinsic metabolic architecture. In this review, we examine how tissue-intrinsic metabolic features shape susceptibility to transformation and the ensuing metabolic rewiring, how the tumor microenvironment imposes additional metabolic constraints, and how this framework informs precision therapeutic strategies. We propose the concept of metabolic permissiveness—the inherent capacity of a tissue to tolerate, adapt to, or exploit metabolic disruptions—as one of several key interacting determinants that shape whether oncogenic mutations can initiate and sustain transformation, with relative contribution varying across tissues and contexts. Finally, we argue that effective metabolism-targeted therapies must account for the tissue-specific metabolic landscape in which transformation occurs rather than pursuing universal metabolic targets.

## Tissue-specific metabolic landscape of cancer: context matters

Different tissues in the body show distinct molecular and metabolic profiles under normal conditions. These baseline tissue-specific differences arise from distinct epigenetic configurations, lineage-defining transcription factors, cellular composition, and microenvironmental cues (soluble factors and neighboring cell types) and reflect each tissue's specialized physiology, developmental origin, hormonal and signaling milieu, and exposure to nutrients and oxygen (Hensley et al. 2016; Schneider et al. 2017; Vander Heiden and DeBerardinis 2017; Gaude et al. 2018; Barata et al. 2022; Peters et al. 2024; Zhang et al. 2024). We briefly describe these in this section and summarize them in Table 1.

**Table 1.**
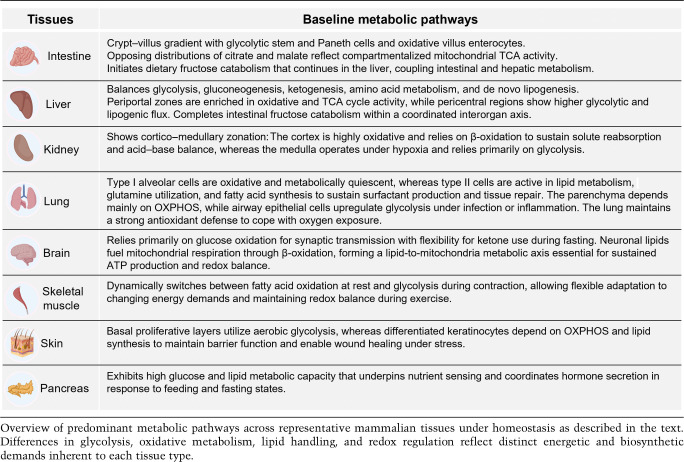
Tissue-specific metabolic features under physiological conditions

The intestine is highly metabolically heterogeneous, with a striking gradient along the crypt–villus axis that mirrors the functional hierarchy of its cell types. Stem cells and Paneth cells residing at the bottom of the crypts primarily rely on glycolysis, while differentiated cells migrating along the villus shift toward OXPHOS and mitochondrial respiration. This compartmentalization supports the energetic and biosynthetic needs of continuous self-renewal and active stem cell cycling while maintaining nutrient absorption, immune regulation, and tight redox control (Cho et al. 2006; Tormos et al. 2011; Berger et al. 2016; Rodríguez-Colman et al. 2017; Goncalves et al. 2019). Beyond epithelial-intrinsic metabolic zonation, gut metabolism cannot be described as purely host-intrinsic, as the local microbiome shapes the baseline metabolic state of the tissue via contributing a diverse range of metabolites. In the intestine (particularly in the colon, where microbial density and metabolite production are highest), epithelial metabolism is further sculpted by microbial-derived compounds, such as short-chain fatty acids (SCFAs), secondary bile acids (SBAs), and tryptophan-derived indoles. SCFAs fuel epithelial oxidative metabolism and act as signaling molecules and epigenetic modifiers, while SBAs and other microbially modified host or dietary compounds engage nuclear and G protein-coupled receptors to influence epithelial redox balance, barrier function, and lineage differentiation (Donohoe et al. 2011; Kaiko et al. 2016; Ornelas et al. 2022). Collectively, these interactions position the gut microbiota as an integral component of host metabolic regulation, such that tissue-specific metabolic permissiveness in the intestine reflects not only epithelial-intrinsic programs but also continuous bidirectional metabolic cross-talk with the resident microbiota that dynamically shapes the baseline metabolic landscape of the mucosa (Tremaroli and Bäckhed 2012).

The liver is a key organ in whole-body energy homeostasis that dynamically balances glycolysis, gluconeogenesis, and ketogenesis while also supporting amino acid catabolism and de novo lipogenesis in response to feeding and fasting states (Rui 2014).

Recent advanced spatial metabolomics analyses have provided high-resolution maps of metabolic zonation, revealing that both the liver lobule and the intestinal epithelium exhibit extensive spatial gradients in metabolite abundance and pathway activity (Samarah et al. 2025). In the liver, periportal enrichment of oxidative, TCA cycle, and amino acid catabolism-related metabolites versus pericentral dominance of glycolytic and lipogenic intermediates has been robustly demonstrated. For the intestine, metabolites such as citrate and malate display opposing crypt–villus distributions, reflecting compartmentalized mitochondrial activity. Notably, this study also delineated the spatial handling of dietary fructose, showing that fructose catabolism initiates predominantly in enterocytes and continues in the pericentral zone of the liver, thereby coupling intestinal and hepatic zonation into a coordinated interorgan fructose metabolism axis (Samarah et al. 2025).

Another organ with pronounced regional metabolic zonation is the kidney. The renal proximal tubule, located in the cortex, demonstrates an exceptionally high reliance on OXPHOS (supported by rich mitochondria content) to sustain active solute reabsorption and acid–base balance. The renal cortex also favors β-oxidation, whereas the medulla, which operates in hypoxia, relies more on glycolysis (Bhargava and Schnellmann 2017; Rabelink and Giera 2019).

Similarly, the lung is continuously exposed to oxygen and environmental stressors—factors that define its redox environment and metabolic flexibility. Type I alveolar epithelial cells are metabolically quiescent, whereas type II cells are highly active in lipid metabolism, glutamine utilization, and fatty acid synthesis to support surfactant production and alveolar repair. The lung parenchyma primarily depends on OXPHOS under steady-state conditions, while airway epithelial cells can upregulate glycolysis in response to infection and inflammation (Rao et al. 2019; Sun et al. 2019; Wang et al. 2024). These examples illustrate that even distinct cell types within the same organ adopt specialized metabolic roles, allowing them to meet diverse physiological demands while maintaining homeostasis.

Other tissues, though less metabolically zonated, exhibit highly specialized metabolic programs tailored to their physiological functions. The brain depends almost exclusively on glucose oxidation to sustain synaptic transmission and ion homeostasis and can shift to ketone body utilization under fasting to supply ATP for cognitive performance (Magistretti and Allaman 2015). However, emerging evidence indicates that neuronal energy metabolism is more flexible than previously recognized. Neuronal lipid droplets are mobilized during increased energy demand, generating free fatty acids (FFAs) that feed directly into mitochondrial metabolism to sustain synaptic function in the brain (Kumar et al. 2025). Complementing this, it has recently been shown that the neuron-specific triglyceride lipase DDHD2 is indispensable for providing the saturated fatty acid flux required for efficient neuronal mitochondrial respiration and ATP synthesis. Disruption of DDHD2 impairs β-oxidation and mitochondrial function even when glycolytic rates are upregulated, therefore evidencing a specific requirement for long-chain fatty acid metabolism within neurons (Saber et al. 2025). Together, these studies reveal a previously underappreciated lipid-to-mitochondria axis that extends beyond the classical view of brain energy metabolism as solely dependent on glucose and ketone bodies.

Skeletal muscles are capable of dynamically shifting between fatty acid oxidation (FAO) at rest and glycolysis during intense activity, thereby enabling rapid energy output for contraction and locomotion (Wang et al. 2017). The stratified epithelium of the skin engages with aerobic glycolysis in basal proliferative layers while utilizing OXPHOS and lipid synthesis in mature keratinocytes to maintain robust barrier function and promote wound healing upon injury (Martic et al. 2023). Last, the pancreas exhibits high glucose and lipid processing capacity to coordinate hormone secretion (insulin, glucagon, and somatostatin) in response to fluctuating nutrient states (Röder et al. 2016).

Cancer appears to act as a metabolic hijacker, rewiring the preexisting metabolic foundations of the tissue of origin rather than creating new pathways from scratch. Pan-cancer transcriptomic analyses support this view. An early integrative study comparing metabolic gene expression across 22 human cancer types and their matched normal tissues revealed that most tumors largely preserve the tissue-specific metabolic gene expression patterns of their origin, with only a subset of pathways, such as glycolysis and nucleotide biosynthesis, being consistently upregulated across cancers. In contrast, alterations in OXPHOS and other metabolic routes were highly variable, reflecting tumor type and context (Hu et al. 2013). Consistent with this, a systematic comparison of metabolic gene expression across 20 solid tumor types from The Cancer Genome Atlas (TCGA) revealed that tumors arising from anatomically or developmentally related tissues tend to share similar metabolic signatures (Gaude and Frezza 2016). These findings argue against a uniform metabolic program in cancer and instead highlight the importance of the tissue of origin to shape the metabolic transformation. Extending this concept, an integrative pan-cancer analysis profiled 95 recurrent driver genes across >9000 primary tumors (Chen et al. 2025). By integrating mutation frequency with transcriptional and epigenomic data from matched normal tissues, it was shown that nearly 80% of driver genes exhibit pronounced tissue specificity, often mirroring their physiological expression domains (Chen et al. 2025). Moreover, tissue-specific and cell type-specific determinants govern oncogenic signaling outputs, emphasizing that the same driver mutation may lead to divergent tumor phenotypes depending on tissue context (Schneider et al. 2017). For instance, it has also been shown recently that metabolic zonation in the liver intrinsically constrains tumor initiation. Using mosaic, zone-specific mutagenesis of canonical hepatocellular carcinoma (HCC) drivers (*Ctnnb1* and *Arid2*), HCC originated predominantly from pericentral (zone 3) hepatocytes despite mutant clones expanding more robustly in periportal (zone 1) regions, demonstrating that spatially encoded metabolic programs define oncogenic competence within the tissue (Guo et al. 2025). These results imply that oncogenes and tumor suppressors must engage in tissue-specific programs to alter metabolism. However, the mechanism by which this occurs is not fully understood. This aspect is discussed in the following section.

## Context-dependent consequences of canonical oncogene activation

Numerous studies have demonstrated that the metabolic outputs of canonical oncogenes and tumor suppressors are not uniformly conserved but are instead dictated by the underlying metabolic architecture of the tissue in which they are either activated or lost. Identical oncogenic mutations can ultimately yield markedly divergent outcomes and tumor phenotypes depending on tissue context. Below, we examine how two of the most frequently mutated oncogenes in human cancer, MYC and KRAS, illustrate these principles, revealing that metabolic rewiring is not a deterministic output of genetic alteration but an emergent property shaped by the metabolic permissiveness of the tissue environment.

### MYC: baseline expression dictates metabolic rewiring

Among oncogenes, MYC acts as a universal transcriptional amplifier of metabolic programs that promote proliferation (Dang 2012; Das et al. 2023). However, its downstream metabolic consequences are profoundly tissue-specific. A recent multitissue transcriptomic and metabolomic analysis revealed that MYC's oncogenic ability is conditioned by its basal expression landscape. Using inducible MYC activation across several murine organs, it has been demonstrated that tissues with high constitutive MYC activity, such as the intestine and skin, exhibit limited transcriptional remodeling and retain metabolic homeostasis, whereas low-MYC tissues like the liver and kidney undergo extensive metabolic reprogramming upon MYC induction. Specifically, low-MYC tissues display pronounced upregulation of glycolytic and nucleotide biosynthetic genes, accumulation of TCA intermediates, and increased mitochondrial biogenesis. These data reveal that baseline transcriptional architecture dictates the metabolic consequences of oncogene activation, explaining why MYC-driven tumorigenesis is tissue-restricted (Popow et al. 2025). In this context, baseline transcriptional architecture refers to the lineage-encoded gene expression programs that define the metabolic identity of a tissue under physiological conditions. These include the constitutive expression of metabolic enzymes and nutrient transporters, the activity of lineage-defining transcription factors, and the epigenetic accessibility of metabolic gene loci, which together determine which metabolic pathways are active, inducible, or even constrained in a given tissue prior to transformation.

### MYC: tissue-specific metabolic programs

Beyond baseline expression, the specific metabolic programs activated by MYC vary dramatically across cancer types, reflecting distinct tissue metabolic environments. In neuroblastoma, MYCN drives glutaminolysis and elevated reactive oxygen species (ROS) generation and has been shown to promote fatty acid uptake and biosynthesis by directly inducing key transporters and enzymes (Wang et al. 2018; Tao et al. 2022). In colorectal cancer (CRC), MYC coordinates widespread metabolic reprogramming by boosting >200 metabolic reactions involving de novo purine and pyrimidine biosynthesis while suppressing mitochondrial biogenesis (Satoh et al. 2017). In triple-negative breast cancer (TNBC), MYC overexpression correlates with enhanced reliance on mitochondrial FAO rather than glycolysis (Casciano et al. 2020). In Burkitt lymphoma, MYC translocation strongly transcriptionally activates pathways tailored to rapid growth and nucleotide biosynthesis in lymphoid cells (Liu et al. 2008). MYC-driven hepatocellular carcinomas exhibit a glutamine-addicted phenotype manifested by repression of glutamine synthetase (GLUL), upregulation of glutaminase (GLS), and severe glutamine depletion that eventually supported rapid proliferation (Yuneva et al. 2012). Strikingly, MYC-driven lung adenocarcinomas (LUADs) display the exact opposite: elevated levels of both enzymes and glutamine accumulation (Yuneva et al. 2012). These data reveal that baseline transcriptional architecture dictates the metabolic consequences of oncogene activation, explaining why MYC-driven tumorigenesis is tissue-restricted.

### KRAS: tissue-specific metabolic adaptations

Turning to KRAS, another key oncogenic driver, its impact on metabolic rewiring exhibits striking context dependence. Consistent with this context dependence, KRAS mutation patterns themselves display marked tissue specificity. Activating KRAS mutations are highly enriched in pancreatic ductal adenocarcinoma (PDAC), CRC, and LUAD yet are rare or absent in many other epithelial tissues, indicating that only selected tissue environments permit both the emergence and metabolic consequences of oncogenic KRAS signaling. In PDAC, the mutant KRAS (*Kras*^*G12D*^) reroutes glucose metabolism toward the nonoxidative pentose phosphate pathway (PPP), hexosamine biosynthesis, and glycosylation processes (Ying et al. 2012). This establishes an alternative noncanonical glutamine utilization circuit involving aspartate transaminase (GOT1) and malic enzyme (ME1) to produce cytosolic NADPH for cellular redox control (Son et al. 2013). Additionally, KRAS drives macropinocytosis to recycle biosynthetic building blocks as an adaptation to the nutrient-deprived PDAC microenvironment and reinforces NRF2-driven antioxidant programs to support proliferation (DeNicola et al. 2011; Kamphorst et al. 2015; Michalopoulou et al. 2020; Liu et al. 2022). Importantly, KRAS-driven macropinocytosis is not uniform across KRAS alleles. The atypical *KRAS*^*G12R*^ mutant, which is relatively enriched in PDAC but rare in other KRAS-driven cancers, shows impaired interaction with the p110α PI3K isoform and reduced engagement of canonical PI3K–AKT signaling, resulting in attenuated KRAS-dependent micropinocytosis. Therefore, compensatory signaling via alternative PI3K isoforms such as PI3Kγ supports nutrient scavenging and highlights allele-specific metabolic dependencies (Hobbs et al. 2020). In contrast, KRAS-driven non-small cell lung carcinoma (NSCLC) preferentially takes up and catabolizes branched-chain amino acids (BCAAs) as a nitrogen source and for protein synthesis, whereas KRAS-driven PDAC shows reduced tumor BCAA uptake, leading to elevated circulating BCAAs in early PDAC (Mayers et al. 2016). In NSCLC, in vivo isotope tracing indicates that glutamine contributes minimally to the TCA cycle anaplerosis, while pyruvate carboxylase (PC) provides essential anaplerotic flux required for lung tumor growth (Davidson et al. 2016). In CRC, oncogenic KRAS primarily upregulates the expression of glucose transporter (GLUT1) and glycolytic genes while also exposing tumors to increased endogenous ROS through the oxidized form of vitamin C (dehydroascorbate [DHA]) that inactivates glyceraldehyde 3-phosphate dehydrogenase (GAPDH) and selectively kills KRAS mutant cells (Yun et al. 2009, 2015). Additionally, KRAS mutant CRC adapts to glutamine depletion by inducing asparagine synthetase (ASNS) via the PI3K–AKT–mTOR signaling pathway to maintain asparagine pools, whereas the dual combination of L-asparaginase and mTOR inhibition by rapamycin could effectively constrain tumor growth (Toda et al. 2016; Doubleday et al. 2021). Finally, KRAS mutant colorectal cancer cells often exhibit de novo lipogenesis driven by sterol regulatory element-binding protein 1 (SREBP1) downstream from PI3K–AKT–mTOR signaling. Selective PI3Kδ inhibitors suppress this pathway and induce ferroptosis, as evidenced by glutathione (GSH) depletion and increased lipid peroxidation in KRAS mutant CRC models (Zheng et al. 2024). These tissue-specific adaptations reveal that KRAS does not impose a universal metabolic program but instead engages distinct metabolic circuits tailored to the nutrient availability and metabolic constraints of each tissue. Recent work further expands the concept of tissue-specific and cell state-specific permissiveness. Oncogenic RAS activation induces rapid phenotypic plasticity and a protumorigenic neutrophil response exclusively in a subset of “permissive” somatic cells. These cells undergo partial dedifferentiation, engage inflammatory signaling, and recruit tumor-promoting neutrophils, creating a microenvironment that facilitates malignant transformation. This confirms that metabolic permissiveness and phenotypic permissiveness are tightly coupled and that oncogene-induced transformation depends not only on tissue context but also on the intrinsic capacity of cells to reprogram their metabolic and phenotypic states (Elliot et al. 2025).

### KRAS: isoform- and allele-specific metabolic heterogeneity

Beyond tissue specificity, distinct KRAS isoforms and allelic variants differentially shape metabolic rewiring. Notably, *KRAS4A*, but not *KRAS4B*, binds directly to hexokinase 1 (HK1) on the outer mitochondrial membrane (OMM) in a GTP- and palmitoylation-dependent manner (Amendola et al. 2019). This interaction relieves allosteric inhibition of HK1 by glucose-6-phosphate, thereby enhancing HK1 enzymatic activity and glycolytic flux, linking oncogenic signaling to the regulation of glycolytic enzymes. In parallel, *KRAS*^*G13D*^ CRC exhibits a unique metabolic program marked by transcriptional and metabolic upregulation of genes involved in glycolysis and serine–glycine and steroid biosynthesis, along with altered amino acid and acylcarnitine abundance profiles. These shifts were accompanied by defective mammalian target of rapamycin complex 1 (mTORC1) and protein kinase B (PKB) activation, as well as transient AMP-activated protein kinase (AMPK) induction upon growth factor stimulation (Charitou et al. 2019). Most strikingly, a recent comparative metabolomic and isotopic tracing study across isogenic CRC lines expressing different *KRAS*^*G12*^ alleles (*G12A*, *G12C*, *G12D*, and *G12V*) uncovered pronounced allele-specific differences in glutamine and nitrogen metabolism. In fact, cells harboring *KRAS*^*G12D*^ and *KRAS*^*G12V*^ mutations show elevated GLUL expression and forkhead box protein O1 (FOXO1)-dependent activation of nitrogen assimilation and recycling pathways. These alterations promoted the incorporation of ammonia into glutamine and enhanced extracellular glutamine secretion, suggesting an adaptive mechanism for nitrogen detoxification and redox buffering. Importantly, these mutants revealed a selective vulnerability to pharmacological inhibition of GLUL and GLS, highlighting a context-dependent metabolic liability. In contrast, *KRAS*^*G12A*^ and *KRAS*^*G12C*^ mutants largely retain canonical glutamine catabolism with minimal FOXO1 activation, underscoring the functional metabolic heterogeneity among different KRAS alleles even within a single tissue context (Ber et al. 2026). This work demonstrates how distinct KRAS mutations can rewire metabolism through discrete regulatory circuits, extending the paradigm of metabolic heterogeneity from the tissue level to the mutational level. Overall, these findings demonstrate that metabolic rewiring induced by canonical oncogenes is influenced by multiple contextual factors, including tissue origin, splice isoform, and mutated allele, which together determine how metabolic networks are remodeled and constrained across tumor types.

## Tumor suppressors and tissue-specific metabolic regulation

Like oncogenes, tumor suppressors such as p53, PTEN, LKB1, and RB1 also regulate metabolic programs whose downstream consequences vary markedly across different cellular environments.

### p53: context-dependent metabolic guardian

*TP53* is the most frequently mutated gene in human cancers, and p53 acts as a multifaceted metabolic gatekeeper whose role is shaped by its wild-type or mutant status. In several tissues, wild-type p53 limits biosynthetic fluxes and subsequently suppresses tumor growth by inhibiting glycolysis and redirecting glucose metabolism toward OXPHOS, thereby reducing reliance on aerobic glycolysis. In addition, p53 supports redox homeostasis and modulates amino acid metabolism and lipid biosynthesis in a context-dependent manner (Liang et al. 2013; Koo et al. 2025). In breast cancer, missense mutant p53 enhances serine and glycine biosynthesis and increases the uptake of essential amino acids via the upregulation of serine synthesis pathway enzymes and the L-type amino acid transporter LAT1/CD98 complex (Tombari et al. 2023). Mutant p53 (gain of function) disrupts normal acinar morphogenesis in breast cancer by upregulating the mevalonate pathway and promoting the transcription of key genes involved in cholesterol and isoprenoid biosynthesis, thereby highlighting the metabolic susceptibility of p53 mutant tumors to perturbations in this pathway (Freed-Pastor et al. 2012). In PDAC, gain-of-function p53 mutant variants such as *p53*^*R270H*^ and *p53*^*R172H*^ distinctly rewire lipid metabolism. The R270H mutant reduces TCA cycle intermediates and alters mitochondrial metabolism and GSH homeostasis, whereas the R172H mutant modulates the urea cycle and acyl-CoA pools, reflecting variant-specific lipidomic remodeling (Caporali et al. 2024; Cotton et al. 2025). In NSCLC, the combination of SH003 (an herbal medicine) and docetaxel chemotherapy impairs de novo pyrimidine biosynthesis by downregulating essential metabolic enzymes, leading to DNA damage and apoptosis. This effect is selective in p53 wild-type NSCLC cells, where activation of p53 enables the apoptotic response to pyrimidine depletion, whereas p53 mutant cells show reduced sensitivity to this metabolic blockade (Choi et al. 2024). In CRC, p53 regulates tumor metabolism by coordinating glycolysis, mitochondrial function, and redox balance in part via its target gene, *TP53*-induced glycolysis and apoptosis regulator (TIGAR). TIGAR acts as a fructose-2,6-bisphosphatase, suppressing glycolysis, redirecting flux into the PPP, enhancing production of NADPH and GSH, lowering ROS, and fostering tumor cell survival (Cheung et al. 2013; Pang and Wu 2025). Overexpression of the mitophagy regulator PINK1 activates p53, resulting in the induction of mitochondrial regulators, enhancing OXPHOS, reducing acetyl-CoA production, and restraining tumor growth. Conversely, PINK1 loss diminishes p53 activity and promotes a glycolytic and protumorigenic metabolic state (Yin et al. 2021).

### PTEN: tissue-specific PI3K–AKT metabolic outputs

Another tumor suppressor gene frequently mutated across several human cancers and associated with radically different metabolic outcomes depending on the tissue of origin is *PTEN*. In the liver, hepatocyte-specific *Pten* deletion causes progressive hepatomegaly, steatohepatitis, and hepatocellular carcinoma (HCC) through sustained AKT activation, which drives lipid synthesis via SREBP-1c and PPARγ. This protumorigenic environment is further favored by elevated ROS, partly resulting from dysregulated peroxisomal β-oxidation coupled with oxidative DNA damage (Horie et al. 2004). In prostate cancer, the combined loss of PTEN and p53 synergistically enhances glycolysis, primarily by increasing hexokinase 2 (HK2) expression and activity. PTEN loss activates the PI3K/AKT/mTORC1 pathway, promoting HK2 mRNA translation, while p53 deficiency stabilizes HK2 mRNA by impairing miR-143 biogenesis. Together, these alterations fuel the Warburg effect, thereby supporting aggressive tumor growth. This metabolic reprogramming contributes to increased glucose uptake, lactate production, and enhanced anabolic metabolism in prostate cancer cells lacking both tumor suppressors (Wang et al. 2014). It has also been reported that PTEN loss results in aberrantly accumulated esterified cholesterol (cholesteryl esters [CEs]) in lipid droplets within prostate cancer cells due to activation of the PI3K/AKT/mTORC1/SREBP pathway (Yue et al. 2014; Ahmad et al. 2021). In ovarian cancer, PTEN restrains glucose uptake by dephosphorylating and thereby limiting AKT-mediated trafficking of GLUT1 to the plasma membrane. Loss of PTEN promotes persistent localization of GLUT1 at the cell surface, thus increasing glucose consumption and supporting tumor growth (Phadngam et al. 2016). Last, in glioblastoma (GBM), PTEN regulates glutamine flux into de novo pyrimidine synthesis, and PTEN-deficient GBM cells show selective sensitivity to inhibition of dihydroorotate dehydrogenase (DHODH), a rate-limiting enzyme in pyrimidine ring synthesis. This was further supported by findings that dual targeting of DHODH and CAD impairs the growth of PTEN-null GMB stem cells and that combined inhibition of receptor tyrosine kinase (AXL) and ribosomal protein S6 kinase 1 (S6K1) suppresses pyrimidine synthesis in PTEN-deficient GBM (Mathur et al. 2017; Pal et al. 2022; Behrmann et al. 2024). Similar patterns of tumor suppressor-driven metabolic rewiring have been reported for LKB1, RB1, and APC in distinct cancer types, each highlighting the importance of tissue context in shaping metabolic dependencies. Although PTEN loss converges on PI3K–AKT signaling across tissues, the dominant metabolic outputs of this pathway, such as glycolysis, lipid synthesis, or nucleotide biosynthesis, are determined by the preexisting metabolic wiring of the tissue, resulting in context-specific dependencies despite shared upstream signaling.

### LKB1: energy stress responses across tissues

LKB1 can activate AMPK, a master metabolic regulator, by direct phosphorylation. Loss of LKB1 promotes increased glucose and glutamine uptake, heightened aerobic glycolysis, and lactate production in LUAD and cervical squamous cell carcinoma. This metabolic shift is mediated via deregulation of the LKB1–AMPK–mTORC1 axis and hypoxia-inducible factor 1α (HIF-1α) stabilization, which eventually drives transcription of glycolytic enzymes such as HK2 and GLUT1 (Shackelford and Shaw 2009; Faubert et al. 2014). In pancreatic cancer models, LKB1 deficiency rewires the serine–glycine–one-carbon (SGOC) pathway to sustain S-adenosyl methionine (SAM)-dependent DNA methylation, thereby mechanistically linking metabolic changes to epigenetically driven tumorigenesis. Importantly, LKB1 loss synergizes with oncogenic KRAS activation to accelerate malignant progression (Kottakis et al. 2016). In HER2-driven breast tumors, LKB1 loss accelerates tumorigenesis and induces a glycolytic shift characterized by increased ATP levels, upregulation of glycolytic enzymes, and hyperactivation of both mTORC1 and mTORC2 signaling (Andrade-Vieira et al. 2013). In LUAD, LKB1 inactivation deregulates key metabolic enzymes such as triosephosphate isomerase 1 (TPI1), thus disrupting the balance between glycolytic flux and lipid biosynthesis (Stein et al. 2023). Furthermore, LKB1-deficient lung tumors rely heavily on autophagy and FAO to maintain metabolic homeostasis under nutrient stress, and simultaneous disruption of these compensatory pathways leads to energy collapse and tumor regression (Bhatt et al. 2019; Bourouh and Marignani 2022).

### Retinoblastoma

Unlike PTEN and LKB1, which primarily interface with nutrient sensing and bioenergetic homeostasis, RB1 indirectly influences metabolism by shaping transcriptional and differentiation programs as well as developmental signaling axes in a context- and tissue-dependent manner. In prostate cancer, RB1 inactivation leads to activation of the transcription factor E2F1 and upregulation of GSH biosynthesis genes and amino acid transporters that enhance the uptake of serine, glycine, and cysteine, fueling the production of GSH and supporting redox buffering capacity under oxidative stress (Mandigo et al. 2021). In the same context, RB1 loss was recently shown to upregulate acyl-CoA synthetase long-chain family member 4 (ACSL4), an enzyme critical for polyunsaturated fatty acid metabolism, via E2F1, rendering RB1-deficient prostate cancer cells selectively vulnerable to ferroptosis, a form of iron-dependent lipid peroxidation cell death (Wang et al. 2023). In *Kras*-driven lung tumors, RB1 deficiency promotes a glycolytic shift manifested by increased glucose uptake via upregulation of key glycolytic enzymes such as GLUT1, HK2, and pyruvate kinase M2 (PKM2) while leaving mitochondrial pyruvate oxidation largely intact (Conroy et al. 2020). In contrast, RB1- and p53-deficient mammary tumor cells in TNBC exhibit increased mitochondrial protein translation (MPT) and elevated OXPHOS, which support tumor growth but also render these cells susceptible to tigecycline, an MPT inhibitor that shows additive effects with sulfasalazine, an inhibitor of cystine import that targets redox vulnerability (Jones et al. 2016). Last, in leiomyosarcoma, RB1 loss represses the expression of phosphoglycerate mutases (PGAM1/2). This reduction in PGAM levels reduces glycolytic metabolite levels and lowers extracellular acidification, thereby diminishing glycolytic flux and impeding differentiation. Interestingly, overexpression of PGAMs or pyruvate supplementation partially rescues differentiation defects and glycolytic activity (Kohno et al. 2025).

Collectively, these examples highlight that tissue context shapes not only how oncogenic and tumor-suppressor mutations reprogram metabolism but also whether such reprogramming can be sustained or converted into a tumorigenic advantage. Complementary evidence indicates that malignant cells can adopt transcriptionally distinct states within the same tumor, each reflecting specific parenchymal lineage features. Such preexisting identity programs shape metabolic dependencies and influence tumor behavior, thereby reinforcing that cellular identity imposes metabolic constraints well before overt transformation or progression (Chalabi Hajkarim et al. 2025; Pei et al. 2025).

## Tissue specificity of TCA cycle gene mutations

While oncogenes and tumor suppressors reveal how the same genetic lesion can rewire metabolism in tissue-specific ways, mutations affecting core metabolic enzymes of the TCA cycle expose the converse principle: identical metabolic defects giving rise to tumors only in select tissues. In fact, some of the most remarkable examples of tissue specificity come from core metabolic enzymes of the TCA cycle, which are traditionally seen as essential and universal yet produce striking tumor-restricted oncogenic effects when mutated, showing that metabolic “housekeeping” functions are indeed context-dependent.

### Fumarate hydratase

Germline mutations in fumarate hydratase (FH), an enzyme that catalyzes the reversible conversion of fumarate to malate, predispose individuals to hereditary leiomyomatosis and renal cell carcinoma (HLRCC), an autosomal dominant cancer predisposition syndrome characterized by benign skin and uterine leiomyomas and a highly aggressive and metastatic form of renal cell carcinoma (Tomlinson et al. 2002; Moch et al. 2022). HLRCC patients inherit one mutant copy of *FH*, while the second allele is lost in tumor tissue via loss of heterozygosity. This represents a typical example of a Knudson two-hit hypothesis of tumor formation, making *FH* a bona fide tumor suppressor gene. Despite its ubiquitous expression and housekeeping role, FH loss leads to malignant transformation only in specific tissues. This paradox suggests that additional tissue-intrinsic factors determine whether FH loss is tolerated and tumorigenic. In epithelial kidney cells, FH loss truncates the TCA cycle, forcing a subsequent switch in carbon utilization. OXPHOS is impaired and mitochondrial glucose entry is restricted by inactivation of the pyruvate dehydrogenase (PDH) complex, while glutamine becomes the primary carbon source for the TCA cycle. Notably, glutamine-derived carbons are diverted toward the heme biosynthesis and degradation pathway, eventually secreting bilirubin (Frezza et al. 2011; Valcarcel-Jimenez and Frezza 2023). One of the most distinctive metabolic hallmarks of FH loss is the accumulation of high millimolar levels of fumarate. This excess fumarate is buffered at least in part by the uptake of exogenous arginine, which generates argininosuccinate via the shifting of the activity of the urea cycle enzyme argininosuccinate lyase, and by free cysteine and glutathione, which bind to fumarate, forming 2-succino-cysteine (2SC) and succinic-glutathione. Crucially, these buffering pathways are essential for the survival of FH-deficient cells, and their inhibition can lead to selective cell death (Bardella et al. 2011a; Sullivan et al. 2013; Zheng et al. 2013, 2023). In parallel with these metabolic shifts, fumarate itself acts as a potent oncometabolite through multiple signaling axes. It stabilizes HIFs, activates NRF2-mediated antioxidant responses via KEAP1 succination, and inhibits α-ketoglutarate–dependent dioxygenases (αKGDs) such as TET enzymes (Sciacovelli and Frezza 2016; Sciacovelli et al. 2016; Tyrakis et al. 2017). In particular, TET inhibition leads to hypermethylation and silencing of the miR-200 family of antimetastatic microRNAs, thereby promoting EMT and invasive behavior (Sciacovelli et al. 2016). Recent work has shown that FH-deficient tumors are selectively sensitive to inhibition of NAMPT, a key enzyme in NAD^+^ salvage, revealing a reliance on this pathway to sustain redox homeostasis in the absence of functional mitochondrial respiration (Najera et al. 2025). However, these oncogenic programs emerge only in cells that survive the initial bioenergetic and redox stress imposed by FH loss. This has led to the proposal of a two-step model of FH-driven tumorigenesis. First, only tissues with sufficient metabolic flexibility and antioxidant buffering capacity can adapt to FH loss and tolerate the fumarate buildup. Second, in these metabolically permissive settings, fumarate accumulation can engage oncogenic signaling pathways that drive transformation and progression. However, experimental evidence supporting this hypothesis remains scant.

### Succinate dehydrogenase

Loss-of-function mutations affecting individual subunits of succinate dehydrogenase (SDH) heterotetrameric complex (SDHA–D) or their assembly factors (SDHAF1–F4) result in the accumulation of succinate, another oncometabolite that competitively inhibits αKGDs, such as prolyl hydroxylases (PHDs), thereby stabilizing HIF-1α and promoting a pseudohypoxic transcriptional program (Baysal et al. 2000; Gottlieb and Tomlinson 2005; Pollard et al. 2005, 2006; Selak et al. 2005; Bardella et al. 2011b). In parallel, it drives global DNA and histone hypermethylation, further contributing to tumorigenesis through epigenetic reprogramming (Killian et al. 2013). These molecular changes have been manifested in several human pathologies, comprising hereditary or sporadic malignancies such as paraganglioma and pheochromocytoma (PPGL/PHEO) that constitute the vast majority of SDH-deficient cancer, rarer cases of gastrointestinal stromal tumors (GISTs), and renal cell carcinomas (RCCs) (Gill et al. 2014; MacFarlane et al. 2020). The striking tissue specificity of SDH mutant tumors, which primarily affect the paraganglionic lineage in PPGL/PHEO, is puzzling. Some suggest that it may come from the unique capacity of these cells to tolerate the metabolic stress caused by SDH loss via their catecholaminergic signaling, innate hypoxia tolerance, and reliance on glycolytic metabolism, allowing SDH-deficient cells to persist and accumulate additional oncogenic alterations that initiate tumorigenesis (Lepoutre-Lussey et al. 2016).

### Isocitrate dehydrogenase

**Figure 1.**
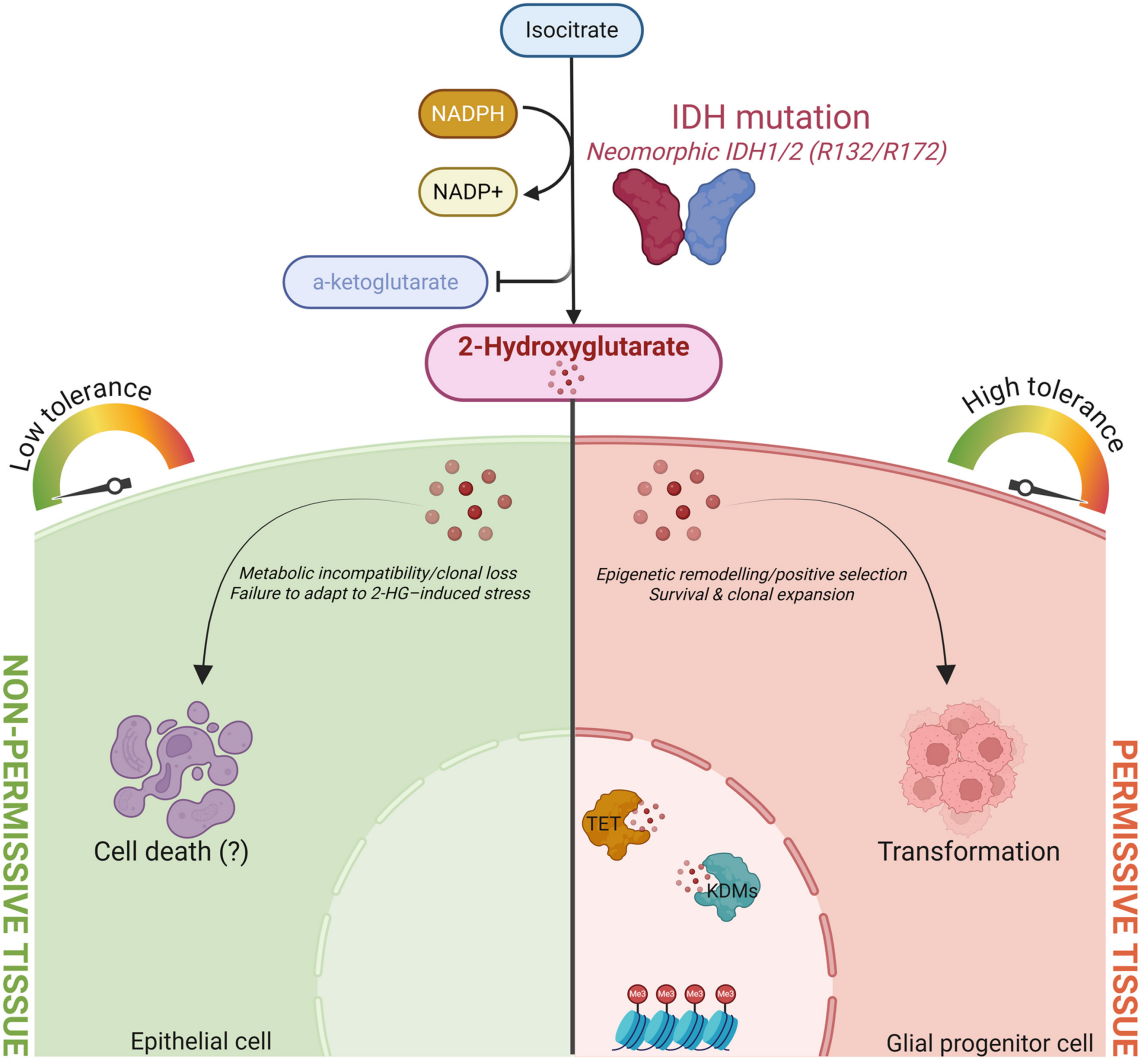
Tissue-specific outcomes of IDH1/2 mutations depend on metabolic permissiveness. Neomorphic mutations in IDH1/2 convert α-ketoglutarate (α-KG) into 2-hydroxyglutarate (2-HG), which inhibits α-KG-dependent dioxygenases such as TET and JmjC demethylases. This inhibition triggers epigenetic remodeling with global DNA and histone hypermethylation and impaired differentiation. The cellular response to this metabolic perturbation is tissue-dependent. In permissive tissues such as the brain, cells tolerate 2-HG-induced stress and undergo epigenetic remodeling that enables positive selection, survival, and clonal expansion, driving transformation. In nonpermissive tissues, including most epithelia, the same perturbation causes metabolic incompatibility or clonal loss due to failure to adapt to 2-HG-induced stress. These contrasting outcomes illustrate how metabolic permissiveness dictates where IDH mutations can initiate tumorigenesis.

Another powerful example of tissue-specific responses to mutation is offered by mutations in isocitrate dehydrogenase (IDH). IDH1 and IDH2 are NADP^+^-dependent enzymes that normally catalyze the oxidative carboxylation of isocitrate to α-KG in the cytosol and mitochondria, respectively. Recurrent mutations in IDH1 (R132) and IDH2 (R140 or R172) confer a neomorphic enzyme activity, producing 2-hydroxyglutarate (2-HG) instead of α-KG (Dang et al. 2009; Ward et al. 2010). 2-HG is a structural analog of α-KG and functions as a competitive inhibitor of αKGDs, including TET family DNA demethylases, JmjC domain histone demethylases, and PHDs. This inhibition leads to widespread epigenetic remodeling, including increased repressive histone methylation and global DNA hypermethylation (Xu et al. 2011; Lu et al. 2012). However, the tissue distribution of IDH mutation-driven tumors is highly specific. IDH1/2 mutations are highly enriched in specific tumor types—particularly lower-grade gliomas and secondary glioblastomas (80%), acute myeloid leukemia (AML; 20%), and central chondrosarcomas—while being rare or absent in most epithelial malignancies. In gliomas, IDH1 mutations are associated with a glioma CpG island methylator phenotype characterized by extensive DNA hypermethylation driven by 2-HG. In AML, IDH2 mutations frequently co-occur with TET2, DNMT3A, and NPM1 and collectively contribute to blocked hematopoietic differentiation and clonal expansion (Yan et al. 2009; Figueroa et al. 2010; Amary et al. 2011; Turcan et al. 2012; Lim et al. 2020). In glioma, IDH1 mutations are typically early events in tumor evolution and are linked to a younger age of onset. Glial progenitor cells are uniquely susceptible to the differentiation-blocking effects of 2-HG yet remain viable long enough to acquire additional oncogenic alterations, sustaining tumor growth over time (Pirozzi and Yan 2021). Even in this case, it is tempting to speculate that cells that tolerate IDH mutations and 2-HG accumulation will eventually undergo transformation. Whether this permissiveness is due to the expression of 2-HG dehydrogenases or the presence of 2-HG “buffering systems” is currently unknown. The tissue-specific consequences of IDH mutation are illustrated in Figure 1.

### Malate dehydrogenase

Malate dehydrogenase (MDH) catalyzes the interconversion of malate to oxaloacetate and exists in two isoforms: the cytosolic MDH1 and the mitochondrial MDH2. Germline *MDH2* mutations have been identified in a small number of patients with PPGL and PHEO yet are absent from large genomic studies of epithelial and mesenchymal cancers, positioning MDH2 as a rare but recurrently altered gene in this tumor type (Calsina et al. 2018). In fact, *MDH2* mutations account for <1% of PPGL cases, in which they are associated with CpG island hypermethylation, reduced 5-hydroxymethylcytosine levels, and transcriptomic shifts consistent with dioxygenase inhibition (Cascón et al. 2015; Remacha et al. 2017). Beyond this rare mutation context, evidence is mounting that MDH1 deregulation occurs in several tumor types, including LUAD, liver, and gastric cancers, and high MDH1 expression correlates with worse prognosis, as indicated by a pan-cancer expression and correlation analysis. Moreover, in glioblastoma, a long noncoding RNA (MDHDH) has been shown to scaffold MDH2 and the proteasome subunit PSMA1, modifying NAD^+^ metabolism and autophagy to suppress tumor growth, thereby suggesting a noncanonical role of MDH1 in the glioma context (He et al. 2022; Lou et al. 2025). That such a central metabolic enzyme is mutated in only a single tumor type but is deregulated across many others highlights the complexity of metabolic constraints in cancer and the importance of cellular context in shaping the consequences of core metabolic disruptions.

### Aconitase

As a final example, the mitochondrial enzyme aconitase 2 (ACO2), which is responsible for the isomerization of citrate to isocitrate, further illustrates how disruption of core metabolic enzymes can be selectively dispensable in certain tumor contexts. Although ACO2 is essential for mitochondrial energy metabolism and broadly expressed, it has not been implicated as a canonical cancer driver gene, and inactivating mutations remain extremely rare. Contrastingly, functional loss or downregulation of *ACO2* may be selectively tolerated or even advantageous in certain tissues. In CRC, ACO2 silencing promotes tumor progression by rerouting citrate-derived carbons toward SCD1-mediated lipid biosynthesis, thus supporting membrane production and cellular proliferation (You et al. 2021). In engrafted aggressive NSCLC, low ACO2 expression enhances tumorigenicity by disrupting mitochondrial iron–sulfur (FeS) cluster maintenance and activating mitochondrial stress signaling pathways. Notably, re-expression of *ACO2* in these cells impairs tumorigenicity in vitro and in vivo (Mirhadi et al. 2023). Similarly, in breast cancer, overexpression of *ACO2* in MCF-7 cells suppresses proliferation by promoting mitochondrial oxidative metabolism and triggering a ROS–FoxO1-mediated autophagic response, reinforcing its tumor-suppressive role in specific epithelial contexts (Ciccarone et al. 2020). Despite these effects, *ACO2* inactivation is not tolerated across tissues. Its downregulation is limited to a subset of tumor types, and naturally occurring *ACO2* loss-of-function mutations have not been observed in epithelial or mesenchymal malignancies. This suggests that in most tissues, complete loss of *ACO2* is either detrimental to cell survival or incompatible with transformation (Wang et al. 2022).

Taken together, the examples of TCA cycle enzyme disruption highlight that even within a pathway considered universal and essential, the effects of metabolic dysfunction are significantly tissue-dependent. Mutations in FH, SDH, IDH1/2, MDH1/2, and ACO2 can drive transformation in specific cellular contexts while being incompatible with survival in others. This variability suggests that each tissue possesses a distinct threshold for tolerating or adapting to mitochondrial dysfunction, a property shaped by its baseline metabolic wiring, redox state, and energetic demands. As we explore in the next section, the tumor microenvironment provides a second layer of metabolic constraints, in which nutrient availability, immune competition, stromal support, and oxygen gradients further shape whether a metabolically compromised cell can survive, adapt, and ultimately transform. The intersection of cell-intrinsic permissiveness and microenvironmental context defines the full landscape of metabolic cancer susceptibility.

## The tumor microenvironment: second layer of tissue-specific constraints

The tumor microenvironment (TME) constitutes a dynamic and critical secondary layer of tissue-specific constraints on cancer metabolism, complementing the effects of cell-intrinsic genetic alterations. While oncogenic mutations reprogram metabolic pathways within cancer cells, the TME exerts additional selective pressures and support mechanisms that vary substantially across tissues. Functioning as a highly adaptive, spatially organized ecosystem, the TME comprises immune populations, stromal and endothelial cells, adipocytes, fibroblasts, and extracellular matrix (ECM) components. All of them interact metabolically and paracrinally with tumor cells, thereby contributing significantly to intratumoral metabolic heterogeneity and influencing tumor progression and organ-specific metastatic tropism. The composition and behavior of the TME differ markedly across tissues, shaped by variations in immune surveillance, vascularization, ECM stiffness, and local oxygen and nutrient availability (Lyssiotis and Kimmelman 2017).

### Immune component of the TME

Immune infiltration is a metabolically significant feature of the TME, and its composition varies markedly by tissue type. Organs such as the liver and gastrointestinal tract are immunologically tolerogenic at baseline due to constant exposure to dietary and microbial antigens. These tissues are enriched in regulatory T cells (Tregs) and macrophages, which shape a suppressive immune landscape even before transformation (Joyce and Fearon 2015; Greten et al. 2019). Consequently, liver tumors often arise within immunosuppressive environments rich in Tregs, myeloid-derived suppressor cells (MDSCs), and alternatively activated macrophages—immune cell types that promote tumor progression by dampening immune surveillance and supporting metabolic adaptations that enhance oxidative stress resistance and immune evasion. In contrast, tumors arising in the lung, skin, or kidney are more likely to emerge within immunologically “hot” microenvironments characterized by high infiltration of cytotoxic T lymphocytes (CTLs), NK cells, and inflammatory macrophages. These settings can initially constrain tumor growth but also impose intense metabolic pressure, selecting for tumor clones with enhanced nutrient uptake, antioxidant defense, or immune checkpoint resistance. Across all tissues, competition for limiting nutrients such as glucose, glutamine, tryptophan (Trp), and arginine is a central metabolic axis of immune–tumor interaction (Chang et al. 2015; Buck et al. 2017; Lim et al. 2020). Effector T cells, particularly CD8^+^ T cells, depend on glycolysis to sustain their cytotoxic activity. However, highly glycolytic tumor cells often deplete glucose within the TME, thereby impairing T-cell function. This nutrient competition not only suppresses antitumor immunity but also selects for metabolically aggressive tumor cells capable of scavenging or synthesizing key nutrients under resource-limited conditions (Chang et al. 2015; Buck et al. 2017; Lim et al. 2020). Beyond direct nutrient competition, immune cells can actively modulate tumor metabolism by secreting cytokines and metabolic enzymes. Tumor-associated macrophages (TAMs) release arginase and nitric oxide synthase (iNOS), thus modulating the local arginine pool and contributing to reactive nitrogen species accumulation (Holmgaard et al. 2015; Groth et al. 2019; Li et al. 2021a; Pataskar et al. 2022). Similarly, MDSCs express indoleamine 2,3-dioxygenase (IDO), leading to Trp depletion and increased kynurenine (Holmgaard et al. 2015; Munn and Mellor 2016; Prendergast et al. 2017). Trp degradation suppresses T-cell function and survival by inducing autophagy, stress responses, and cell cycle arrest (Pataskar et al. 2022), while kynurenine serves as an endogenous ligand for the aryl hydrocarbon receptor (AhR) in tumor cells, promoting gene expression relevant to immune evasion and metabolic adaptation (Ball et al. 2009; Munn and Mellor 2016; Prendergast et al. 2017). These effects are more pronounced in lymphoid-rich environments such as the gut and lymph nodes, where T-cell fitness is highly sensitive to tryptophan availability (Munn and Mellor 2016). Moreover, the depletion of extracellular arginine by arginase-expressing myeloid cells promotes urea cycle rewiring in tumor cells, a phenomenon especially prominent in lung and colorectal cancers, where altered arginine metabolism supports anabolic growth and resistance to oxidative stress (Holmgaard et al. 2015; Groth et al. 2019; Li et al. 2021a,b; Pataskar et al. 2022).

### The TME stroma

The nonimmune stromal compartment of the TME, primarily including cancer-associated fibroblasts (CAFs), adipocytes, and stellate cells, delivers tissue-specific paracrine signals and metabolic substrates that profoundly reshape tumor metabolism. Among these, CAFs represent one of the most abundant noncancerous cell populations within the TME and undergo pronounced metabolic reprogramming that increases the production and release of high-energy metabolites, including lactate, pyruvate, and alanine. These metabolites are subsequently taken up by adjacent tumor cells, where they serve as critical substrates for OXPHOS and anaplerosis. Such metabolic coupling between CAFs and tumor cells exemplifies a dynamic, symbiotic metabolic relationship that highly supports tumor progression (Pavlides et al. 2009; Zhang et al. 2015; Li et al. 2021b). In PDAC, stroma-associated pancreatic stellate cells (PSCs) provide nonessential amino acids (NEAAs), specifically alanine, to tumor cells via autophagic degradation to fuel TCA cycle anaplerosis (Sousa et al. 2016). In breast cancer, glycolytic CAFs export lactate via monocarboxylate transporter 4 (MCT4), providing oxidative tumor cells with an alternative fuel source. This lactate shuttle sustains OXPHOS in tumor cells, promoting their survival and metastasis (Martinez-Outschoorn et al. 2014; Affinito et al. 2024). In adipose-rich microenvironments such as the breast, ovary, or peritoneal cavity, adipocytes release FFAs via increased lipolysis, which are taken up by cancer cells to boost FAO and membrane biosynthesis. This metabolic cross-talk is amplified in obesity and hypoxic conditions, where cancer-associated adipocytes (CAAs) undergo phenotypic changes, including reduced lipid stores and altered adipokine secretion, further promoting tumor progression and therapy resistance (Wen et al. 2017; Koundouros and Poulogiannis 2020; Mukherjee et al. 2022). Similarly, CAFs in CRC undergo lipid metabolic reprogramming characterized by elevated fatty acid synthase (FASN) expression and increased secretion of fatty acids and phospholipids. This CAF-driven lipogenic shift, mediated by IL-6/IL-11–STAT3 signaling, promotes tumor proliferation, angiogenesis, and a protumorigenic microenvironment in CRC (Gong et al. 2020; Heichler et al. 2020). At the same time, recent pan-cancer spatial transcriptomic analyses have identified conserved CAF subtypes and associated cellular neighborhoods across multiple solid tumors independent of tissue of origin, linking stromal organization to immune infiltration and clinical outcomes and indicating that tissue-specific CAF metabolic programs are layered onto a shared stromal architecture (Liu et al. 2025).

### The extracellular matrix

The ECM also varies in structure and composition across tissues, thereby further shaping tumor metabolism (Pickup et al. 2014). ECM stiffness and topology influence mechanotransduction pathways such as YAP/TAZ, PI3K–Akt, and integrin–FAK signaling, which in turn regulate glycolysis, lipid metabolism, and redox balance (Mouw et al. 2014; Rubashkin et al. 2014; Cooper and Giancotti 2019). Dense collagen-rich ECMs, such as those found in the pancreas or desmoplastic breast tumors, impair diffusion of glucose and oxygen, generating metabolic gradients that tumor cells must adapt to (Winkler et al. 2020; Yuan et al. 2023). Therefore, cancer cells upregulate nutrient-scavenging pathways like macropinocytosis or matrix degradation via matrix metalloproteinases (MMPs) to liberate matrix-bound growth factors and nutrients and facilitate invasion (Kessenbrock et al. 2010; Commisso et al. 2013). In contrast, brain tumors grow within a proteoglycan-rich yet relatively soft ECM. This unique ECM environment, coupled with local hypoxia, has been associated with lipid droplet formation and rewiring of lipid metabolism in glioblastoma, including enhanced lipogenesis and context-dependent engagement of FAO (Guo et al. 2009; Bensaad et al. 2014).

### Oxygen and nutrients shape the TME

Hypoxia and nutrient gradients are additional hallmark features of the TME, and their degree and spatial heterogeneity vary strongly across tumor types and tissue contexts. In chronically hypoxic regions, the HIF-1α and HIF-2α subunits accumulate in the nucleus, where they dimerize with the aryl hydrocarbon receptor nuclear translocator (ARNT) and activate hypoxia-responsive elements. Then, they drive transcriptional programs that upregulate glycolytic enzymes and promote angiogenesis via vascular endothelial growth factor (VEGF) secretion (Rankin and Giaccia 2016). The interplay between vascular architecture and ECM structure thereby imposes tissue-specific metabolic constraints. In tissues where vessels are denser and the ECM is less restrictive, tumor cells may preferentially adopt oxidative or biosynthetic programs, whereas in tissues with poor perfusion or a stiff ECM, selection favors glycolysis, nutrient scavenging, or alternative metabolic routes.

The TME also facilitates direct metabolic cross-talk with noncancerous parenchymal cells. In the omentum, metastatic ovarian cancer cells co-opt resident adipocytes, inducing lipolysis and thus releasing FFAs, which are taken up by tumor cells to fuel β-oxidation and sustain rapid proliferation and growth (Nieman et al. 2011). In the brain, GBMs and brain metastasis adapt to a nutrient-poor, low-glucose environment by oxidizing acetate as an alternative bioenergetic substrate. This adaptation is mediated by upregulation of the enzyme acyl-CoA synthetase short-chain family member 2 (ACSS2), which converts acetate to acetyl-CoA for use in the TCA cycle, sustaining ATP production and lipid biosynthesis. Notably, high ACSS2 expression correlates with tumor aggressiveness and poor prognosis, revealing a potential metabolic vulnerability exploitable in therapy (Mashimo et al. 2014).

Taken together, this layered complexity of the TME underscores the importance of considering both local and systemic factors when studying cancer metabolism and metastatic progression. The immune landscape, stromal interactions, vascular architecture, ECM composition, and parenchymal cell cross-talk all differ strongly across tissues and can contribute to the overall metabolic fitness of the cancer cell.

Tissue context continues to impose strong metabolic constraints during metastatic colonization and outgrowth. Although primary tumors and their metastases frequently share the same driver mutations, successful dissemination requires cancer cells to adapt their metabolism to the nutrient availability, oxygen tension, and redox state of the target organ (Piskounova et al. 2015; Hensley et al. 2016). Importantly, metastatic cells do not fully reset their metabolic identity but instead retain elements of a lineage-imprinted “metabolic memory” inherited from the primary tumor while selectively acquiring niche-driven adaptations that enable growth in distant tissues. These adaptive programs can be strongly shaped by redox limitation during dissemination and colonization (Piskounova et al. 2015), as well as by target organ-specific rewiring of central carbon metabolism, including increased pyruvate carboxylase-dependent anaplerosis during lung colonization (Christen et al. 2016). At the same time, metabolic adaptation during metastatic progression is tightly coupled to epigenetic and transcriptional regulation, with metabolites such as acetyl-CoA, α-KG, and SAM shaping chromatin accessibility and gene expression programs that support outgrowth in distant tissues (Kaelin and McKnight 2013; Pavlova and Thompson 2016; Sun et al. 2022). Together, these studies support the view that metastatic outgrowth reflects a balanced interplay between tissue of origin constraints and organ-specific microenvironmental selection, extending tissue context as a determinant of cancer metabolism beyond primary tumor growth (Sivanand et al. 2024).

## Targeting cancer metabolism: context matters, too

Our review proposes that metabolic rewiring in cancer is not a uniform program but a highly contextual process shaped by tissue identity, tumor stage, microenvironmental inputs, and evolutionary history. These factors not only determine whether specific oncogenic mutations can initiate transformation but also define which metabolic pathways become essential for tumor maintenance and progression. While targeting metabolism in cancer has long been proposed as a promising approach, most metabolism-targeted therapies do not work universally. Numerous therapeutic strategies have been developed to exploit metabolic dependencies in cancer, including small-molecule inhibitors such as IDH1, GLS, or monocarboxylate transporter (MCT1) inhibitors, as well as broader approaches like dietary interventions (low-serine or ketogenic diets), nutrient depletion therapies (arginine or asparagine depletion), and the modulation of immune cell metabolism (targeting enzymes like arginase or IDO1). While each of these approaches is mechanistically sound, their effectiveness has proven to be highly context-dependent (Luengo et al. 2017; Stine et al. 2022).

IDH1/2 inhibitors (ivosidenib and enasidenib, respectively) have demonstrated remarkable efficacy in subsets of AML and gliomas, where 2-HG induces the epigenetic reprogramming essential for tumor maintenance. Its reduction limits tumor burden in responsive patients. However, cholangiocarcinomas and chondrosarcomas, which harbor identical mutations, exhibit inconsistent clinical responses due to tissue-specific differences in 2-HG detoxification capacity, chromatin states, or cellular differentiation programs (Okoye-Okafor et al. 2015; Stein et al. 2017; Mellinghoff et al. 2021). Similarly, GLS inhibitors like CB-839 (telaglenastat) showed promising preclinical activity in TNBC and RCC but failed to succeed in lung or pancreatic cancer. These failures reflect that some tumors develop ways to bypass their dependency on glutamine by either enhancing PC, increasing autophagy, or receiving stromal glutamine support (Gross et al. 2014; Biancur and Kimmelman 2018). MCT1 inhibitors, such as AZD3965, block lactate exchange between the tumor and the stroma. This not only disrupts redox homeostasis but has also been shown to inhibit lipid biosynthesis and increase immune cell infiltration in preclinical models. Despite being a promising strategy, its success depends on tumor reliance on MCT1, which highly varies by tissue type and the presence of compensatory transporters like MCT4 (Beloueche-Babari et al. 2020).

Arginine-depleting enzymes (ADI-PEG20) are among the most effective nutrient depletion therapies in ASS1-deficient mesothelioma and hepatocellular carcinoma but are largely unsuccessful in tumors capable of re-expressing ASS1 or scavenging arginine from the extracellular environment. Mechanistically, arginine starvation induces mitochondrial and metabolic distress in ASS1-negative cells but is ineffective in tumors that can synthesize arginine internally via ASS1 (Cheng et al. 2018; Sun and Zhao 2022). However, prolonged arginine deprivation can also exert selective pressure on tumor populations, leading to the re-expression of ASS1 in metastatic tumor clones, conferring therapeutic resistance and posing significant challenges for the durability of response. The re-expression of ASS1 has been shown to occur through transcriptional activation mechanisms, including c-Myc-dependent regulation, highlighting adaptive metabolic plasticity in cancer cells under nutrient stress. Therefore, strategies combining arginine deprivation with approaches that prevent or counteract ASS1 re-expression may be necessary to achieve effective and sustained tumor control (Kremer et al. 2017; Sun and Zhao 2022; Feng et al. 2025). Even dietary interventions show highly variable outcomes. Serine- and glycine-restricted diets impair growth in colorectal tumors with poor biosynthetic capacity but are inadequate in pancreatic cancers that upregulate PHGDH (Maddocks et al. 2017). Ketogenic diets, proposed to starve glycolytic tumors, suppress gliomas in some models but can promote aggressiveness in others, especially those with strong oxidative or lipid metabolism (Dal Bello et al. 2022). Newer strategies, such as modulating immune cell metabolism, have also been applied. Inhibiting lactate production or transport can reinvigorate antitumor immunity in some settings but can exacerbate immune exhaustion or autoimmunity in others, depending on local immune composition and nutrient competition (Angelin et al. 2017; Pérez-Tomás and Pérez-Guillén 2020).

Collectively, these examples point to a precision metabolic therapy that considers tissue-specific metabolic wiring, tumor stage and progression history, microenvironmental characteristics, interorgan metabolic cross-talk, and co-occurring mutations or epigenetic alterations. Without integrating these parameters, clinical trials risk failing not because the target is irrelevant but because the context is ignored.

## Outlook: cancer through the lens of metabolic permissiveness

**Figure 2.**
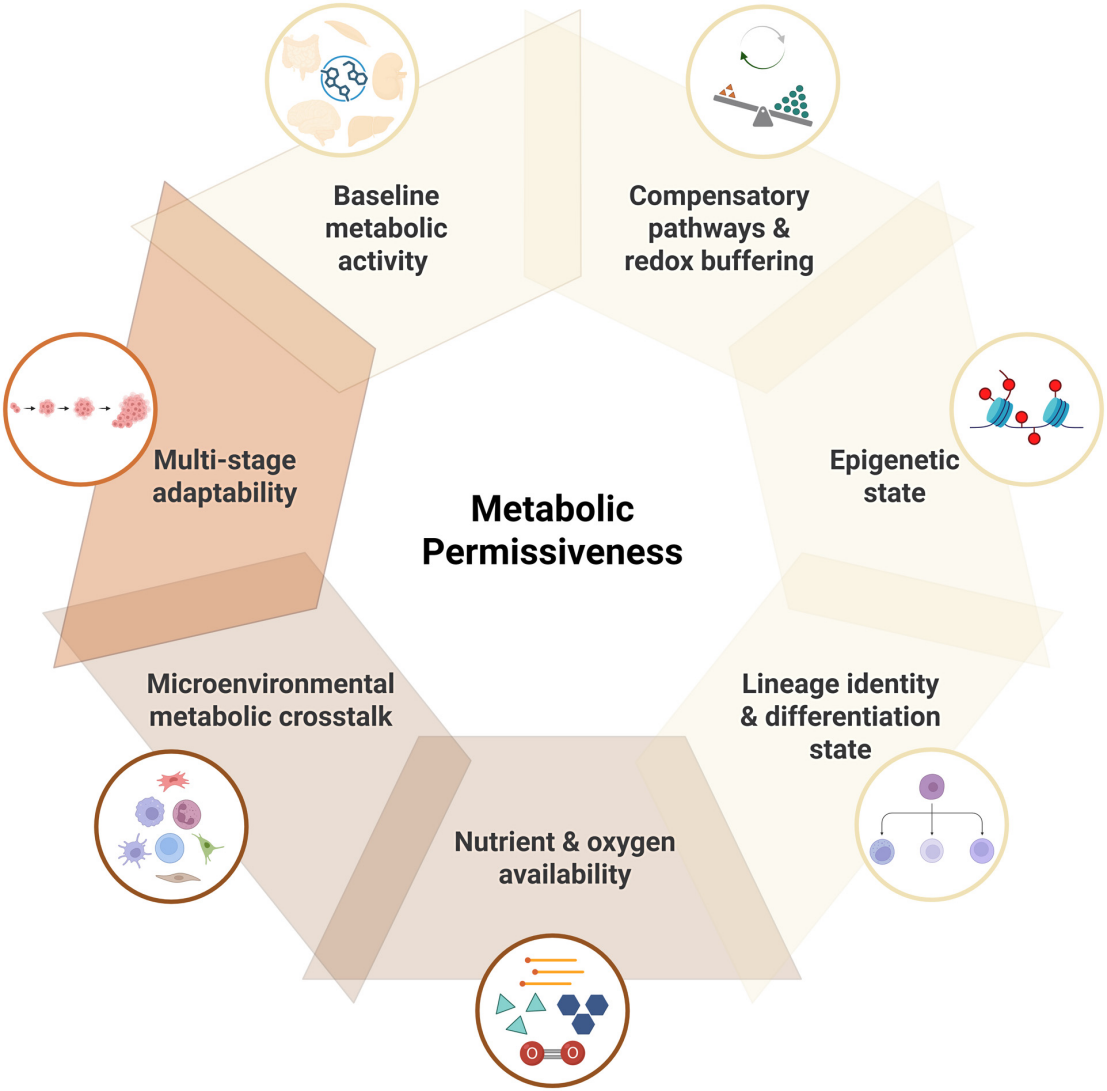
The key principles of metabolic permissiveness across tissues. Metabolic permissiveness reflects the ability of a tissue to tolerate, buffer, or exploit oncogenic metabolic perturbations. It arises from the interplay of intrinsic (light yellow), extrinsic (light brown), and dynamic (dark brown) determinants. Intrinsic factors, including baseline metabolic activity, epigenetic state, lineage identity, and redox buffering capacity, define the cell-autonomous metabolic architecture. Extrinsic factors, such as nutrient and oxygen availability and microenvironmental metabolic cross-talk, define the environmental context, whereas the dynamic multistage adaptability represents the capacity to adjust metabolism during tumor evolution. Together, these hallmarks shape tissue-specific metabolic fitness landscapes that determine where oncogenic mutations can drive transformation.

In this review, we provide evidence that identical oncogenic mutations result in distinct metabolic changes depending on the tissue in which they occur. In addition, we show that they may enhance proliferation and survival in one tissue but may impose excessive metabolic stress or fail to confer a selective benefit in another. Therefore, whether a mutation gives rise to cancer in specific tissues may depend on each tissue's intrinsic capacity to buffer, tolerate, or exploit the metabolic perturbations that it induces, a concept we define as “metabolic permissiveness.” In permissive contexts, cells can survive the initial metabolic derangement caused by somatic mutations. In nonpermissive tissues, the same mutations may impose a fitness cost resulting in a metabolic crisis or triggering programmed cell death and therefore are purged through negative selection. For example, KRAS mutations thrive in the pancreas (where high autophagy and macropinocytosis buffer metabolic stress) but are rare in the brain (where neurons cannot tolerate bioenergetic disruption). Similarly, MYC amplification drives lymphomas and liver cancers (tissues with high biosynthetic capacity) but is selected against in postmitotic muscle (where anabolic demand cannot be met). We propose that the tissue's baseline metabolic activity, nutrient availability, epigenetic state, lineage program, redundant or compensatory pathways, and redox buffering capacity determine whether a given mutation is tolerated and confers a selective advantage or instead leads to clonal extinction. This overarching concept is depicted in Figure 2, with metabolic permissiveness positioned centrally and the associated hallmarks arranged around it. Critically, TME features interact with cell-intrinsic metabolic permissiveness to determine whether genetically altered cells can survive, adapt, and progress, creating a tissue-specific metabolic fitness landscape that extends beyond the cancer cell itself. This two-layer model explains why some mutations are oncogenic only in specific tissues (e.g., IDH1 in glia but not hepatocytes and VHL mostly in kidneys) and why metastatic success depends on metabolic compatibility between tumor cells and distant organ niches. In this framework, tissue context not only shapes the metabolic reprogramming of transformed cells but also acts as a metabolic gatekeeper, where mutations can emerge and persist during early tumorigenesis. This gatekeeper function operates at multiple stages: (1) mutation tolerance (whether the cell can survive the immediate metabolic perturbation), (2) clonal expansion (whether the mutation confers a fitness advantage in this tissue's metabolic niche), (3) microenvironmental adaptation (whether the TME can provide metabolic support), and (4) metastatic seeding (whether the distant organ permits metabolic adaptation). Failure at any stage results in clonal extinction, explaining why most mutations remain “passengers” in most tissues but become “drivers” in metabolically permissive ones.

Notably, this framework applies not only to mutations in metabolic enzymes, such as FH or SDH, but also to all other known oncogenes and tumor suppressors whose mutations are likely to influence metabolism. The broad applicability of this principle is clear in various contexts: TP53 mutations modify glucose and serine metabolism, RAS mutations reroute glutamine use and reprogram macropinocytosis, loss of PTEN boosts glycolysis and lipid synthesis, and VHL mutations lead to pseudohypoxia and changes in glycolytic activity. In each scenario, the metabolic effects, rather than the genetic changes themselves, determine whether the mutation is tolerated in a specific tissue. This logic is further reinforced by hereditary cancer syndromes caused by germline mutations in which the initiating lesion is present systemically, yet tumor development remains highly tissue-restricted. For example, germline *TP53* mutations in Li-Fraumeni syndrome confer a strong predisposition to adrenocortical carcinoma, while germline *APC* mutations in familial adenomatous polyposis predominantly drive intestinal tumorigenesis, and *BRCA1/2* mutations preferentially give rise to breast and ovarian cancers despite systemic loss of gene function (Fearon 2011; Malkin 2011; Roy et al. 2012; Bougeard et al. 2015). More speculatively, these metabolic alterations, often considered to be “driven” by oncogenes, may also be adaptive responses to their mutations, whose compatibility with tissue-specific metabolic constraints ultimately dictates oncogenic outcome.

Metabolic permissiveness is not a binary trait. Rather, it represents a tissue-specific continuum shaped by multiple intrinsic and extrinsic factors, encompassing both the tissue's capacity to adapt to oncogene-induced metabolic stress and the presence of tissue-specific resistance or defense mechanisms that actively limit transformation. From this perspective, cancer susceptibility reflects both the ability of some tissues to adapt to metabolic perturbations and the capacity of others to actively resist them. A highly oxidative tissue, such as the kidney or muscle, may be more vulnerable to mitochondrial dysfunction than a glycolytic epithelium, such as the colon or skin, which could buffer such perturbations more effectively. Conversely, the redox sensitivity and epigenetic plasticity of neural tissues may explain why glial cells are particularly susceptible to 2-HG. Beyond local tissue and microenvironmental constraints, systemic metabolic state can further modulate tissue-specific permissiveness. Conditions such as visceral obesity, insulin resistance, and metabolic syndrome are associated with altered nutrient availability, hormonal signaling, and chronic low-grade inflammation, all of which have been linked to increased cancer risk across multiple tissues (Calle and Kaaks 2004; Doyle et al. 2012; Scully et al. 2021). In the liver, metabolic dysfunction-associated steatotic liver disease (MASLD) exemplifies how systemic metabolic alterations can reshape the tissue metabolic landscape and lower the threshold for malignant transformation in part by cooperating with underlying genetic and epigenetic changes (Faubert et al. 2020; Younossi and Henry 2021; Karra et al. 2022). Recognizing metabolic permissiveness as a continuum influenced by both tissue-intrinsic properties and systemic metabolic state reframes our approach to cancer metabolism—from assuming universal metabolic hallmarks to a contextual framework grounded in tissue identity and physiological constraints. It links the selective pressures acting during tumor initiation, microenvironmental adaptation, and metastatic progression to a common underlying principle. This perspective offers a powerful conceptual bridge between metabolism, tissue biology, and oncogenesis and not only helps to explain the striking tissue specificity of certain cancer-associated metabolic mutations but may also guide therapeutic efforts to exploit tissue-specific metabolic vulnerabilities (Gaude and Frezza 2016; Vyas et al. 2016; Mayers and Vander Heiden 2017; Kreuzaler et al. 2020).
